# Supplementary material for: Anti-Inflammatory Effects of Cannabigerol in Rheumatoid Arthritis Synovial Fibroblasts and Peripheral Blood Mononuclear Cell Cultures Are Partly Mediated by TRPA1
Source: Int J Mol Sci. 2023 Jan 3;24(1):855. doi: 10.3390/ijms24010855 (PMC9820932; doi:10.3390/ijms24010855)
Supplement: Supplementary file 1 [file ijms-24-00855-s001.zip › ijms-2096593-supplementary.pdf]

Supplementary Materials

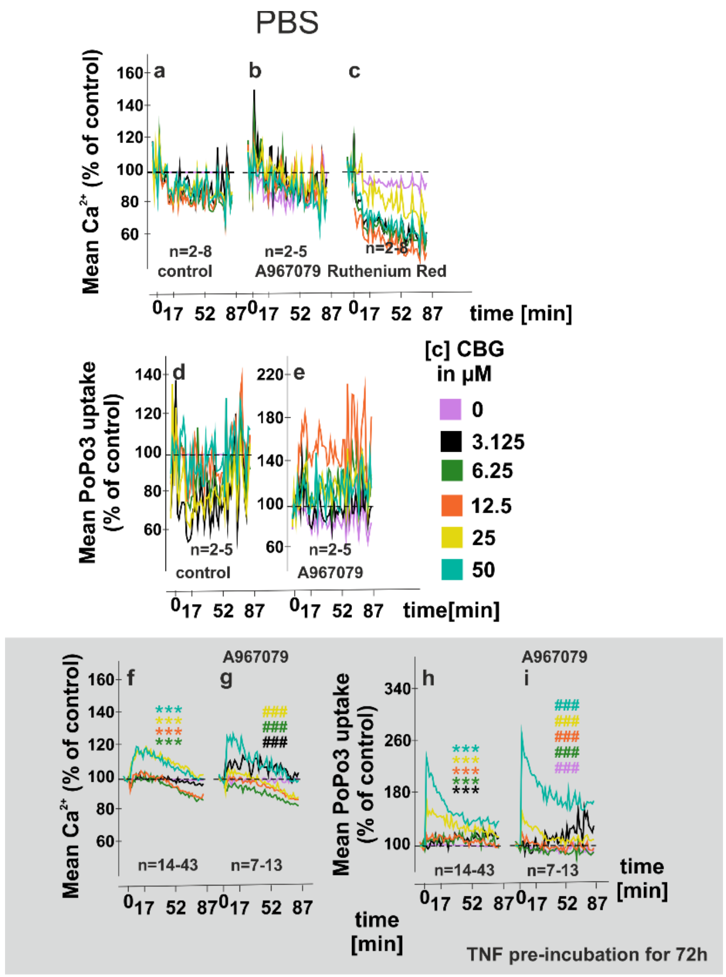

Figure S1. Intracellular calcium levels and PoPo3 uptake of RASF under the influence of CBG and without extracellular calcium (PBS). Influence of CBG on intracellular calcium levels (**a**–**c**;**f**,**g**) **and PoPo3 uptake** (**d**,**e**,**h**,**i**). RASF were simulated (**f**–**i**) or not (**a**–**e**) with TNF for 72h and assays were conducted without extracellular calcium by using phosphate buffered saline (PBS). The TRPA1 inhibitor A967079 or pan TRP inhibitor ruthenium red were added 30min prior to the addition of CBG. *** $p < 0.001$ for differences between concentrations of CBG. ANOVA with Dunnett's T3 post-hoc test was used for comparisons. ### $p < 0.001$ for comparisons of CBG versus CBG/antagonist treatment at each concentration. ANOVA with Bonferroni post-hoc test was used for comparisons. Number of patients included: (**a**) n = 2 and n = 8 (no CBG); (**b**–**e**) n = 2 and n = 5 (no CBG); (**f**,**h**) n = 14 and n = 43 (no CBG) (**g**,**i**) n = 7 and n = 13 (no CBG).

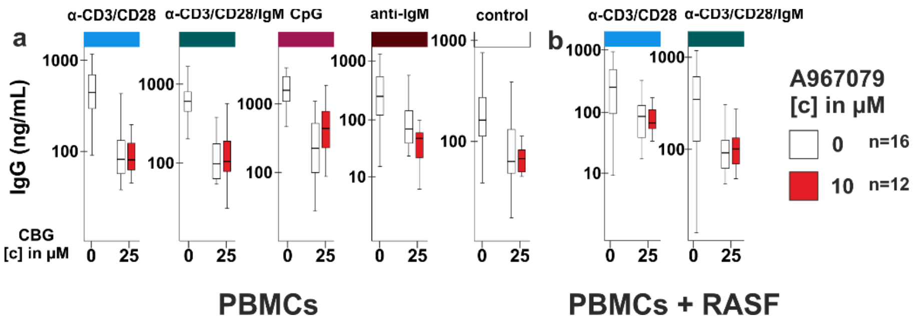

Figure S2. TRPA1 involvement in Immunoglobulin G (IgG) production modulated by CBG in PBMCs and PBMC/RASF co-cultures. (**a**,**b**) PBMCs alone (**a**) or PBMC/RASF co-cultures (**b**) were co-incubated with CBG [25 μM] (controls are shown for comparison) and the TRPA1 inhibitor A967079 [10 μM] under different activation stimuli for 7 days. IgG levels are presented on a logarithmic scale. There was no modulation of IgG production by the TRPA1 antagonist A967079.

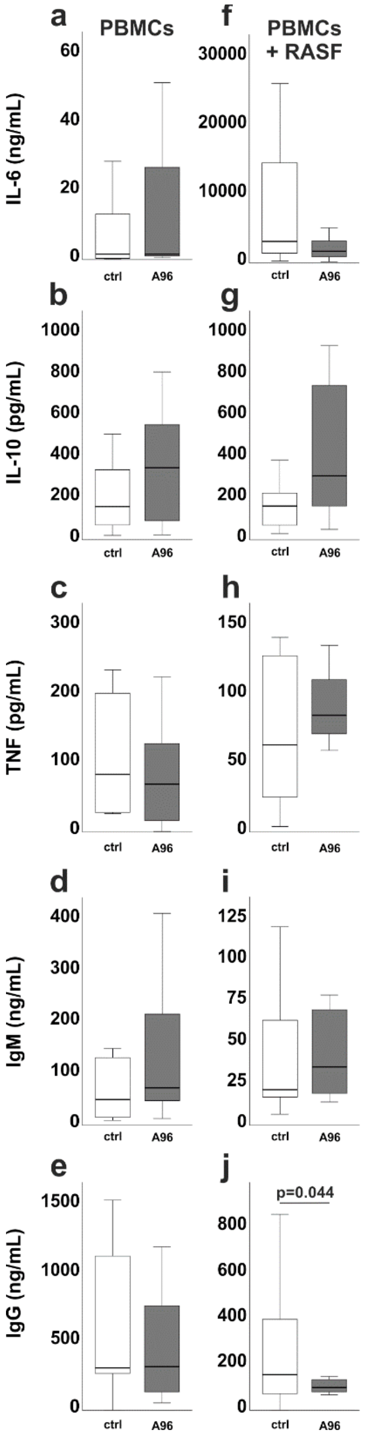

Figure S3: Influence of TRPA1 inhibition on IL-6, IL-10, TNF, IgM and IgG production by PBMCs and PBMC/RASF co-cultures. Influence of the TRPA1 inhibitor A967079 [10 μM] without CBG was assessed in 7 day cultures of PBMCs (**a**–**e**) and PBMC/RASF co-cultures (**f**–**j**). $p < 0.05$ for differences between treatment with A9607079 versus control. Mann Whitney U-test was for comparisons. Gray = A967079 [10 μM], white = control.
